# Supplementary material for: Hybrid Models and Biological Model Reduction with PyDSTool
Source: PLoS Comput Biol. 2012 Aug 9;8(8):e1002628. doi: 10.1371/journal.pcbi.1002628 (PMC3415397; doi:10.1371/journal.pcbi.1002628)
Supplement: Text S4 — Complete source code for the PyDSTool package (version 0.88.120504). Includes API documentation and help files linking to web pages. This file is identical to the current public release on Sourceforge.net. (ZIP) [file pcbi.1002628.s004.zip › PyDSTool/html/PyDSTool.Toolbox.optimizers.step.restart_conjugate_gradient-module.html]

xml version="1.0" encoding="ascii"?


PyDSTool.Toolbox.optimizers.step.restart\_conjugate\_gradient


| Home | Trees | Indices | Help | PyDSTool |
| --- | --- | --- | --- | --- |

Package PyDSTool ::
Package Toolbox ::
Package optimizers ::
Package step ::
Module restart\_conjugate\_gradient

[hide private]

[frames] | no frames]

# Module restart\_conjugate\_gradient

source code

Restarts a conjugate gradient search by deleting the step key in the
state dictionary


|  |  |
| --- | --- |
| Classes | [hide private] |

RestartPeriodicallyConjugateGradientStep  
A step decorator that periodically deletes the direction key in the
dictionary so that the CG search can be restarted

RestartNotOrthogonalConjugateGradientStep  
A step decorator that deletes the direction key in the dictionary
if the last gradients are not orthogonal enough so that the CG
search can be restarted

| Home | Trees | Indices | Help | PyDSTool |
| --- | --- | --- | --- | --- |

|  |  |
| --- | --- |
| Generated by Epydoc 3.0.1 on Fri May 4 15:24:05 2012 | http://epydoc.sourceforge.net |
